# Editorial: Recent advancements and new developments in reconstructive surgery

**DOI:** 10.3389/fsurg.2026.1807999

**Published:** 2026-04-10

**Authors:** Joseph M. Escandón, Chihiro Matsui, Pedro Ciudad, Oscar J. Manrique

**Affiliations:** 1 Department of Surgery, Wyckoff Heights Medical Center, New York, NY, United States; 2 Department of Plastic and Reconstructive Surgery, Juntendo University School of Medicine, Tokyo, Japan; 3 Ciruesthetic Clinic, Reconstructive and Burn Surgery, Lima, Peru; 4 Austin-Weston Center for Cosmetic Surgery, Reston, VA, United States

**Keywords:** advancement, innovation, microsurgery, plastic surgery, reconstructive surgery

Recent advancements and new developments in reconstructive surgery are of paramount importance as the field continues to evolve at the intersection of innovation, patient-centered care, and multidisciplinary collaboration (1). Reconstructive surgery plays a critical role not only in restoring form and function after trauma, oncologic resection, and congenital anomalies, but also in improving quality-of-life and psychosocial well-being (2, 3). Rapid progress in microsurgery, perforator flap design, lymphatic surgery, nerve regeneration, biomaterials, and regenerative medicine has expanded the reconstructive armamentarium and enabled procedures that were previously considered unattainable or associated with significant morbidity (4, 5).

These innovations are reshaping surgical decision-making by promoting more tailored, less invasive, and function-preserving approaches. Given the rapid pace of progress, it is essential to periodically reassess emerging techniques, technologies, and concepts that are redefining contemporary reconstructive practice (6). Highlighting recent developments not only informs clinicians of evolving standards of care but also identifies current limitations and future directions, ultimately fostering innovation that translates into safer procedures, improved functional recovery, and better patient-reported outcomes (7, 8).

The articles included in this collection encompass a broad spectrum of contemporary advances in reconstructive surgery, spanning methodological innovation, large-scale utilization analyses, and evolving procedural techniques. Topics addressed include, but are not limited to, stem cell–based and regenerative therapies; integrated multidisciplinary pathways for the management of lymphedema; lower extremity reconstruction and limb salvage; perforator flap surgery; hand surgery and complex upper-extremity reconstruction; complex abdominal wall reconstruction; and advances in lymphatic surgery and breast reconstruction.

In addition, this collection highlights emerging technologies and novel applications, such as the integration of artificial intelligence in microsurgical planning and intraoperative decision-making, innovations in oculoplastic and craniofacial reconstruction—including the use of xenografts for facial defects—and developments in genital reconstructive surgery. Collectively, these contributions reflect the breadth, technical sophistication, and rapidly evolving nature of modern reconstructive practice.

At the translational frontier, the experimental study by Targosinski et al. evaluating local vs. systemic adipose-derived stromal cell (ASC) therapy in peripheral nerve regeneration provides important insights into regenerative strategies for nerve injury. Using a standardized rodent sciatic nerve transection model, the authors demonstrate that ASC therapy modestly enhances functional recovery, reduces muscle atrophy, and supports axonal remyelination when compared with controls. Notably, systemic administration showed a slight trend toward earlier and superior functional recovery relative to local delivery, suggesting that the therapeutic effects of ASCs may extend beyond localized application through homing and systemic paracrine mechanisms. While the observed differences were modest, this work highlights critical unanswered questions regarding optimal delivery routes, dosing, timing, and potential synergistic strategies combining local and systemic approaches. As peripheral nerve reconstruction remains a major challenge in reconstructive and peripheral nerve surgery, such preclinical data are essential to guide future translational and clinical investigations.

The management of secondary extremity lymphedema (SEL) represents another domain where innovation is driven by an improved understanding of disease pathophysiology. The review by Chen et al. proposing an integrated surgical treatment model based on algorithms addresses the limitations of isolated physiological or excisional approaches. By synthesizing current evidence and introducing the ISTL algorithm, this work emphasizes a component-based, individualized framework that accounts for lymphatic function, disease stage, and tissue composition. The highlighted “3L” strategy—combining lymphaticovenous anastomosis, vascularized lymph node transfer, and liposuction—illustrates how hybrid approaches can more effectively address both the fluid and solid components of lymphedema. This contribution is particularly important in promoting standardized, reproducible decision-making in a field historically characterized by heterogeneity in indications and outcomes, and it reinforces the growing role of microsurgical lymphatic reconstruction within comprehensive care pathways.

Several studies in this collection focus on microsurgical reconstruction for complex defects, particularly in challenging clinical settings such as chronic infection, extensive tissue loss, and oncologic recurrence. The retrospective analysis by Cao et al. of the free chimeric medial sural artery perforator (cMSAP) flaps for chronic osteomyelitis of the foot and ankle demonstrates reliable infection control, durable soft tissue coverage, and improved limb function without the need for secondary debulking procedures. These findings support the cMSAP flap as a valuable option for reconstructing deep dead space and complex wounds, where both contour and function are critical. In a similar vein, the comparative study by Li et al. of local vs. free flap reconstruction for lower limb bone exposure wounds provides clinically relevant data to guide flap selection. While local flaps were associated with fewer complications and shorter operative times, free flaps yielded superior functional recovery and quality-of-life outcomes at one year. This balanced analysis reinforces the importance of tailoring reconstructive strategies to patient priorities, defect characteristics, and institutional expertise rather than adhering to a one-size-fits-all approach.

The evolving role of free flaps in reconstructive surgery is further contextualized by Yin et al. through their bibliometric analysis examining global research trends from 2004 to 2025. By identifying key thematic areas—namely, expanding clinical applications and the integration of computer technologies with biomaterials and surgical planning—this study provides a macroscopic view of where the field is headed. The increasing intersection between microsurgery, digital technology, and material science underscores the transition toward data-driven and precision-based reconstruction. Such analyses are valuable not only for researchers and trainees but also for clinicians seeking to align practice with emerging evidence and innovation.

Innovation at the level of technique and incision design is exemplified by the case report by Shi et al., which describes functional thumb reconstruction in a child with mirror hand deformity. Congenital mirror hand remains one of the most complex challenges in pediatric hand surgery, requiring meticulous planning to achieve both functional and aesthetic goals. The authors’ novel palmar-based incision and thoughtful selection of the dominant digit for pollicization enabled restoration of thumb opposition and adduction while preserving intrinsic musculature and minimizing visible scarring. This case highlights how incremental technical refinements can have substantial functional implications, particularly in rare congenital conditions where standardized approaches are lacking.

Complex abdominal wall reconstruction is addressed in two compelling case reports that illustrate the versatility and necessity of flap-based solutions in extreme scenarios. As described by Li et al., the reconstruction of a massive abdominal wall defect following recurrent colon cancer using bilateral thoracoabdominal flaps and a vertical rectus abdominis myocutaneous flap underscores the critical role of multidisciplinary collaboration. Beyond restoring structural integrity, the reconstruction facilitated meaningful improvements in quality-of-life for a patient previously debilitated by disease. Complementing this, the report by Shen et al. of an adult congenital omphalocele managed with a free anterolateral thigh flap combined with fascia lata grafts demonstrates an innovative autologous solution for large defects without reliance on synthetic materials. The authors achieved durable abdominal wall strength, acceptable aesthetics, and preservation of fertility potential, highlighting the adaptability of microsurgical techniques even in rare and anatomically complex presentations.

Minimally invasive and endoscopic techniques represent another important theme across this collection. Chen et al. reported the endoscopy-assisted excision of a giant axillary cystic lymphangioma. The authors illustrate how enhanced visualization can facilitate precise dissection through smaller incisions, reducing morbidity while achieving complete resection. Similarly, the experience with endoscopy-assisted total mastectomy with and without immediate breast reconstruction demonstrates that oncologic safety can be preserved while improving aesthetic outcomes, reducing surgical trauma, and enhancing patient satisfaction. The favorable Breast-Q and Scar-Q outcomes reported with endoscopic approaches in this study by Lin et al., reflect a broader shift toward value-based surgery, where functional, aesthetic, and psychosocial outcomes are increasingly prioritized alongside traditional surgical metrics.

Advances in lymphatic reconstruction are exemplified by Pan et al., who reported a case of staged, skin-derived vascularized lymph node transfer (VLNT) combined with liposuction for the management of bilateral lower-extremity lymphedema. Bilateral disease remains particularly difficult to manage, and the described two-stage approach—utilizing distinct donor sites and combining physiological reconstruction with excisional therapy—illustrates a thoughtful, tailored strategy. The substantial volume reduction, sustained improvement in lymphatic function on imaging, and durable quality-of-life benefits over long-term follow-up reinforce the growing consensus that integrated approaches targeting both fluid and solid components of lymphedema yield superior outcomes. This case further underscores the expanding role of skin-derived and alternative lymph node donor sites in minimizing donor-site morbidity while maintaining therapeutic efficacy.

The importance of early, anatomically informed intervention is further emphasized by Adi et al. in the report and review of congenital midline cervical cleft (CMCC), a rare but potentially debilitating developmental anomaly. By synthesizing embryological insights with clinical and histopathological features, the authors highlight the risk of progressive contracture and functional limitation if CMCC is left untreated. The successful application of Z-plasty in a neonatal patient reinforces this technique as the reconstructive gold standard, effectively restoring neck contour and mobility while preventing recurrence. The discussion of emerging genetic associations and future regenerative strategies positions CMCC management within a broader framework of precision and personalized surgery.

Innovation at the intersection of technology and reconstruction is a central theme in the review of advances in autologous breast reconstruction. As breast cancer incidence continues to rise globally, the demand for durable, natural, and patient-centered reconstructive solutions has intensified. Abdominal-based free flaps remain the cornerstone of autologous reconstruction, yet this work by Scherrer et al. emphasizes how preoperative imaging, intraoperative navigation, and artificial intelligence are reshaping surgical planning, perforator selection, and risk stratification. These developments signal a paradigm shift toward data-driven microsurgery, with the potential to enhance safety, efficiency, and patient satisfaction while reducing donor-site morbidity.

Oncologic reconstruction of delicate anatomical regions is addressed in the study by Xie et al., who evaluated xenogeneic acellular dermal matrix (XADM) combined with full-thickness skin grafts for eyelid reconstruction following malignant tumor excision. Eyelid defects pose unique functional and aesthetic challenges, requiring precise restoration of both anterior and posterior lamellae. The favorable outcomes reported—including reliable graft integration, minimal complications, and satisfactory functional recovery—support XADM as a valuable reconstructive adjunct. This approach expands the reconstructive armamentarium in oculoplastic surgery, particularly for large, full-thickness defects where local tissue options may be limited.

The review of tension-relieving suturing techniques highlights a fundamental yet often underappreciated principle of surgical reconstruction: the management of mechanical forces at wound edges. By tracing the evolution of tension-reduction strategies and introducing the concept of super–tension-relieving sutures using barbed materials, Ge et al. emphasize how biomechanical optimization can directly influence healing quality, scar formation, and long-term outcomes. The applicability of these techniques across multiple surgical specialties underscores their broad clinical relevance and potential to standardize improved wound care practices.

Finally, innovation in genital reconstruction is illustrated by the clinical study by Li et al., who evaluated the five-flap technique for posterior fourchette reconstruction. Addressing both functional impairment and sexual dysfunction, this approach demonstrates significant improvements in pain, sexual function, and aesthetic outcomes with a low complication rate. The results highlight the importance of patient-reported outcomes in reconstructive surgery and reinforce the role of tailored flap design in managing complex vulvar deformities.

Collectively, these and other studies in our special topic highlight the dynamic and multidisciplinary nature of modern reconstructive surgery. From stem cell–based therapies and algorithm-driven lymphatic reconstruction to advanced microsurgical flaps, minimally invasive techniques, and patient-reported outcome measures, the field continues to expand its scope and sophistication. Importantly, these contributions emphasize that progress in reconstructive surgery is not defined by a single innovation but by the thoughtful integration of biology, technology, surgical technique, and individualized care (9). As reconstructive surgeons are increasingly called upon to address complex defects in medically fragile and oncologic patients, such evidence-based, patient-centered approaches will remain central to advancing outcomes and quality of life.
